# Supplementary material for: ChatGPT as a Tool for Biostatisticians: A Tutorial on Applications, Opportunities, and Limitations
Source: Stat Med. 2025 Oct 23;44(23-24):e70263. doi: 10.1002/sim.70263 (PMC12548020; doi:10.1002/sim.70263)
Supplement: Supplementary file 1 — Data S1. Supporting Information. [file SIM-44-0-s001.zip › Supplementary Materials Additional.zip!/3.7_Translation_Between_Multiple_Statistical_Programming_Languages/3.7.2_SPSS_to_R/3.7.2_Translation_Chat8.pdf]

R 8

Clear memory

I can't clear memory myself, but you can do it by following these steps:

1. Go to **Settings**.
2. Click on **Personalization**.
3. Select **Memory**.
4. From there, you can view and delete specific memories or clear all of them.

Let me know if you want help navigating to it!

Take the role of an expert biostatistician for this chat

Absolutely — I'll take on the role of an expert biostatistician for this chat. Feel free to ask me anything related to study design, statistical modeling, clinical trials, data analysis in biomedical research, survival analysis, longitudinal data, or anything else in the realm of biostatistics. What are you working on?

I am running the following analysis in SPSS:
DATASET ACTIVATE DataSet5. LOGISTIC REGRESSION VARIABLES V2 /METHOD=FSTEP(LR) V3 V4 V5 V6 V7 V8 V9 V10 V11 V12 V13 V14 V15 V16 V17 V18 V19 V20 V21 V22 V23 V24 V25 V26 V27 V28 V29 V30 V31 V32 /CRITERIA=PIN(0.05) POUT(0.10) ITERATE(20) CUT(0.5).
Translate this into R code.
The data is loaded into R with
temp <- tempfile()
download.file(
 "https://archive.ics.uci.edu/static/public/17/breast+cancer+wisconsin+diagnostic.zip",
 temp
)
data <- read.table(unz(temp, "wdbc.data"), sep = ",")
data$V2 <- data$V2 == "M"
unlink(temp)

Great — you're running a **forward stepwise logistic regression** in SPSS using likelihood ratio tests with entry/removal thresholds of 0.05 and 0.10 respectively.

Here's how to translate that analysis into R, assuming you've already read the data and recoded `V2` as a binary outcome:

1. Rename variables for clarity (optional but recommended)

```r
# Assign column names (from the UCI description)
colnames(data) <- c("ID", "Diagnosis", paste0("V", 3:32))
data$Diagnosis <- as.numeric(data$Diagnosis)  # TRUE (M) = 1, FALSE (B) = 0
```

2. Run logistic regression with stepwise selection

R's `step()` function does AIC-based stepwise selection, **not** p-value based like SPSS, but for a closer match, we can use the `MASS::stepAIC()` function. To match SPSS's **forward stepwise (LR)** using **p-values**, we'll use the `stepwise()` function from the `olsrr` or `glmulti` package, or write a custom approach.

A. Closest analog using `MASS::stepAIC()` for forward selection

```r
library(MASS)

# Null model (intercept only)
null_model <- glm(Diagnosis ~ 1, data = data, family = binomial)

# Full model with all predictors
full_model <- glm(Diagnosis ~ ., data = data[, -1], family = binomial)  # remove ID column

# Forward stepwise selection
model_step <- stepAIC(null_model,
                      scope = list(lower = null_model, upper = full_model),
                      direction = "forward",
                      trace = TRUE)
summary(model_step)
```

B. Closer match using p-values with `olsrr` package (optional)

```r
# install.packages("olsrr")
library(olsrr)

# Run logistic regression with forward stepwise selection
model <- glm(Diagnosis ~ ., data = data[, -1], family = binomial)
ols_step_forward_p(model, prem = 0.05, pstay = 0.10, details = TRUE)
```

> 🧠 **Note**: SPSS uses *likelihood ratio tests* for stepwise regression. In R, using `stepAIC()` is a common alternative, though it uses AIC for model comparison. If you're looking for exact LR test analogs with p-values, a custom stepwise function using `anova(..., test = "LRT")` would be needed.

Would you like help writing a fully customized LR-based stepwise function in R that exactly mimics SPSS's behavior?

Yes

Perfect — here's how to build a **custom forward stepwise logistic regression** in R using **likelihood ratio tests (LRT)** and **p-values**, mimicking the behavior of your SPSS command:

🔧 Custom Forward Stepwise Logistic Regression Function (LRT-based)

```r
forward_stepwise_lr <- function(data, outcome, predictors, pin = 0.05, pout = 0.10, max_iter = 20) 
  selected_vars <- c()
  remaining_vars <- predictors
  iter <- 0
  current_model <- glm(as.formula(paste(outcome, "~ 1")), data = data, family = binomial)

  while (length(remaining_vars) > 0 && iter < max_iter) {
    iter <- iter + 1
    pvals <- sapply(remaining_vars, function(var) {
      test_model <- glm(as.formula(paste(outcome, "~", paste(c(selected_vars, var), collapse = "+")
                        data = data, family = binomial)
      lr_test <- anova(current_model, test_model, test = "LRT")
      lr_test$`Pr(>Chi)`[2]  # p-value of added variable
    })

    best_var <- remaining_vars[which.min(pvals)]
    best_p <- min(pvals, na.rm = TRUE)

    if (!is.na(best_p) && best_p < pin) {
      selected_vars <- c(selected_vars, best_var)
      remaining_vars <- setdiff(remaining_vars, best_var)
      current_model <- glm(as.formula(paste(outcome, "~", paste(selected_vars, collapse = "+"))),
                           data = data, family = binomial)
      message(sprintf("Step %d: Added %s (p = %.4f)", iter, best_var, best_p))
    } else {
      break
    }
  }

  # Optionally remove non-significant predictors based on POUT
  if (length(selected_vars) > 0) {
    final_model <- current_model
    repeat {
      pvals <- summary(final_model)$coefficients[-1, 4]
      worst_p <- max(pvals)
      if (worst_p > pout) {
        remove_var <- names(which.max(pvals))
        selected_vars <- setdiff(selected_vars, remove_var)
        final_model <- glm(as.formula(paste(outcome, "~", paste(selected_vars, collapse = "+"))),
                           data = data, family = binomial)
        message(sprintf("Removed %s (p = %.4f)", remove_var, worst_p))
      } else {
        break
      }
    }
    return(final_model)
  } else {
    return(current_model)
  }
}
```

🧪 Usage with your breast cancer data

r

```r
# Assume data is already loaded and cleaned
data$Diagnosis <- as.numeric(data$Diagnosis)  # TRUE (M) = 1, FALSE (B) = 0
predictors <- paste0("V", 3:32)  # SPSS V3–V32
model <- forward_stepwise_lr(data = data, outcome = "Diagnosis", predictors = predictors)
summary(model)
```

✅ What this does:

- Mimics SPSS's `` `/METHOD=FSTEP(LR)` `` using **likelihood ratio tests**.
- Uses:
  - `` `PIN(0.05)` `` — variable enters if LRT p < 0.05
  - `` `POUT(0.10)` `` — variable removed if p > 0.10 after inclusion
- Stops at 20 iterations or when no variable qualifies.

Would you like to add model performance evaluation next (e.g., ROC AUC, confusion matrix, calibration plot)?
